# Simulation Analysis and Optimization Design of Dome Structure in Filament Wound Composite Shells

**DOI:** 10.3390/polym17101421

**Published:** 2025-05-21

**Authors:** Yuan Zhou, Yuyang Zou, Qingguo Xia, Longkai Cao, Minghua Zhang, Tao Shen, Jianke Du

**Affiliations:** Zhejiang-Italy Joint Lab for Smart Materials and Advanced Structures, School of Mechanical Engineering and Mechanics, Ningbo University, Ningbo 315211, China; 18771936583@163.com (Y.Z.); zyy342900250@163.com (Y.Z.); 2201090019@nbu.edu.cn (Q.X.); 15719260805@163.com (L.C.); zhangminghua@nbu.edu.cn (M.Z.)

**Keywords:** carbon fiber-reinforced composites, pressure vessel, dome reinforcement, finite element analysis, structural optimization

## Abstract

Carbon fiber-reinforced composites are widely used in the aerospace industry due to their exceptional mechanical properties. However, the dome region of composite pressure vessels is prone to stress concentrations under internal pressure, often resulting in premature failure and reduced burst strength. This study developed a finite element model of a reinforced dome structure, which showed excellent agreement with hydrostatic test results, with less than 5.9% deviation in strain measurements. To optimize key reinforcement parameters, a high-accuracy surrogate model based on a backpropagation neural network was integrated with a multi-objective genetic algorithm. The results indicate that compared to the unreinforced dome, the optimized structure reduced the maximum fiber-aligned stress in the dome region by 6.8%; moreover, it achieved a 9.3% reduction in overall mass compared to the unoptimized reinforced configuration. These findings contribute to the structural optimization of composite pressure vessel domes.

## 1. Introduction

Carbon fiber composites, consisting of carbon fiber reinforcements and resin matrices, are widely used in aerospace and defense due to their high strength-to-weight ratio, thermal stability, and design versatility [1,2,3]. These advantages have made filament wound carbon fiber shells the preferred choice over metal structures in solid rocket motors, where they serve as primary load-bearing components. However, their high stiffness and sensitivity to stress concentrations present structural challenges, particularly at geometric transitions and material interfaces. Under extreme loading conditions (e.g., hydrostatic bursting), failures typically initiate at weak points, such as dome openings and metal–composite joints, which significantly reduce the fiber strength utilization in cylindrical sections [4,5,6].

Recent studies have proposed various reinforcement methods to enhance the load-bearing capacity of composite shells, particularly for end dome structures [7,8,9]. Liu Bingyu et al. [10] experimentally validated reinforcement methods to effectively mitigate metal ejection and optimize stress distribution in composite pressure vessels across varying diameters. Wang Dong’s comparative analysis [11] revealed that hoop carbon cloth reinforcement provided the most substantial burst pressure enhancement among three evaluated techniques. While these studies have effectively enhanced the structural stability of end domes, the reinforcement methods predominantly rely on empirical judgment, lacking quantitative analysis and theoretical optimization, often resulting in structural redundancy and increased weight.

With the advancement of finite element modeling, artificial intelligence, and multi-objective optimization technologies, researchers have increasingly incorporated numerical simulations and optimization algorithms into reinforcement design. Wang Xin et al. [12] proposed a predictive model for dome thickness distribution using cubic splines, validating the accuracy of high-precision dome modeling and reinforcement strategies. Ramirez et al. [13] developed a Type IV pressure vessel model using WCM plugin, validating its burst prediction accuracy. Soykok et al. [14] systematically evaluated winding angle effects on dynamic structural performance, establishing an optimal fiber layup configuration. Furthermore, Deng et al. [15] employed neural networks combined with the bat algorithm to optimize process parameters, significantly improving design efficiency and structural performance. In the optimization of key design variables, such as initial reinforcement radius, the number of layers and fiber bandwidth have rarely been reported, which motivates the present work.

This study optimized dome reinforcement for a ϕ 300 mm filament wound carbon fiber composite shell. A high-fidelity finite element model was first developed based on grid theory layup design and its accuracy was validated through hydrostatic test data. Key design variables including reinforcement initiation radius, the number of layers, and fiber bandwidth were then used to build a neural network surrogate model. This model was coupled with a multi-objective genetic algorithm to determine the optimal reinforcement combination. The goal was to improve stress distribution and structural reliability in the dome while meeting lightweight design requirements, offering theoretical and methodological guidance for high-performance composite shell design.

## 2. Calculation of Filament Winding Patterns

### 2.1. Grid Theory

**Figure 1:**
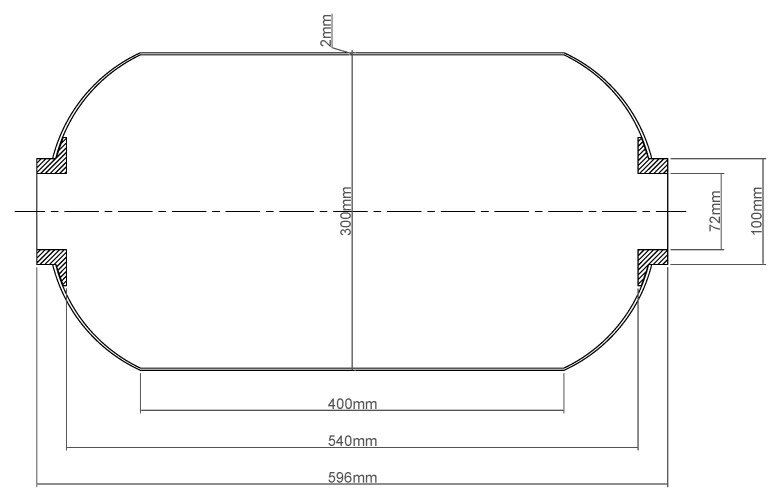
Dimensions of the core mold of the composite shell.

In engineering practice, shell layup design is typically guided by grid theory. In this study, a 300 mm diameter shell with equal polar openings was adopted, and its mandrel geometry is illustrated in Figure 1. The dome featured an elliptical profile with a polar opening diameter of 100 mm, a cylindrical section length of 400 mm, and an overall shell length of 596 mm.

According to grid theory, the fiber thicknesses of the longitudinal and hoop layers in the cylindrical section of the shell could be calculated using the following formulas [16,17]:

(1) $$h_{\varphi }=\frac{RP}{2k_{s}\sigma_{f}cos^{2}\alpha }$$

(2) $$h_{\theta }=\frac{RP}{2\sigma_{f}}\left(2-\frac{1}{k_{s}}tan^{2}\alpha \right)$$

In Equations (1) and (2), $h_{\varphi }$ and $h_{\theta }$ denote the thicknesses of the longitudinal and hoop fiber layers, respectively; *R* is the radius of the cylindrical section, and *P* is the design burst pressure. $\sigma_{f}$ represents the effective fiber strength, which was set to 1800 MPa in this study; α denotes the winding angle of the longitudinal fibers in the cylindrical section; and $k_{s}$ is the stress balance coefficient. To reduce the redundant mass associated with the introduction $k_{s}$, it was assigned a value of 1 in this work, without compromising the accuracy of the calculations.

Given that the shell mandrel featured an equal polar opening configuration, the geodesic winding angle could be determined using the following formula [18,19]:

(3) $$\theta \left(R\right)=sin^{-1}\left(\frac{R_{0}}{R}\right)$$

In Equation (3), *R* denotes the radial distance from the longitudinal winding layer to the axis of rotation and $R_{0}$ represents the radial distance from the transition point to the axis of rotation. This formula is used to calculate the winding angle along the geodesic path for shells with the same polar opening structures.

By associating Equations (1) and (2), we obtained the formula for the total fiber thickness of the case barrel section:

(4) $$h_{f}=h_{\varphi }+h_{\theta }=\frac{(2k_{s}+1)RP}{2k_{s}\sigma_{f}}$$

Based on the above fiber thickness calculation, we further obtained the formula for the final burst pressure of the case:

(5) $$P=\frac{2k_{s}h_{f}\sigma_{f}}{(2k_{s}+1)R}$$

### 2.2. Composite Shell Design

Based on the previous formula and parameter values, the design of the barrel section, and the stacking sequence shown in Table 1, the design burst pressure of the case was 19 MPa, with six helical layers and six hoop layers [20].

The specific sequence of winding and reinforcement operations for the composite shell is shown in Table 2. During the winding process, four bundles of carbon fibers were used. The bandwidth of the helical layer was 8.8 mm and the bandwidth of the hoop layers was 8.1 mm. The thickness of the winding layer in the cylinder section was 2.4 mm and the helical winding angle was 19.5°. To increase the strength of the shell, three reinforcement operations were carried out using T700 plain weave carbon fabric (Guangwei Composites Co., Ltd., Weihai, China).

## 3. Finite Element Analysis and Experiment

### 3.1. Finite Element Modeling

**Figure 2:**
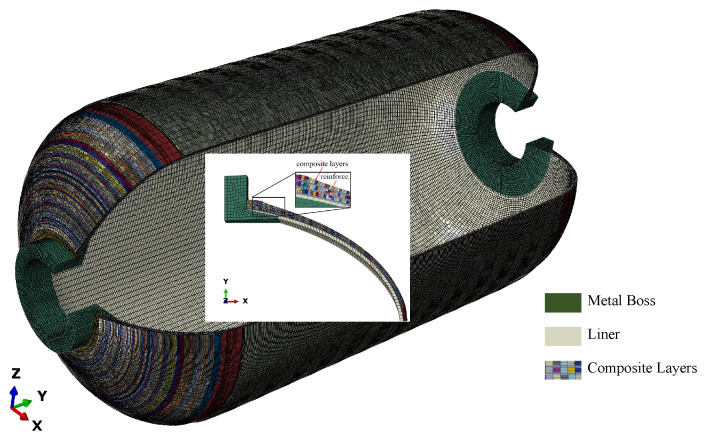
Refined finite element model of composite shell.

The finite element model of the composite shell, considering geometric nonlinearity, was established using ABAQUS software (Dassault Systèmes, Vélizy-Villacoublay, France), as shown in Figure 2. To address the complex variation in angle and thickness in the head region, the winding angle was discretized in 0.5° increments and the mesh was refined in the transition region to capture the continuous variation in angle and thickness [21].

The finite element shell structure was primarily composed of a metal boss, rubber lining, T800 fiber winding composite layers, and T700 dome reinforcement. The metal boss was made of 30CrMnSiA forging, the insulation layer was made of EPDM rubber, and the carbon fiber composite consisted of T800 carbon fiber (Guangwei Composites Co., Ltd., Weihai, China) and HCM-2184 epoxy resin (Ningbo Shuxiang Composite Materials Co., Ltd., Ningbo, China). The performance parameters of the metal boss, rubber lining, and carbon fiber composite are listed in Table 3 and Table 4, respectively.

**Figure 3:**
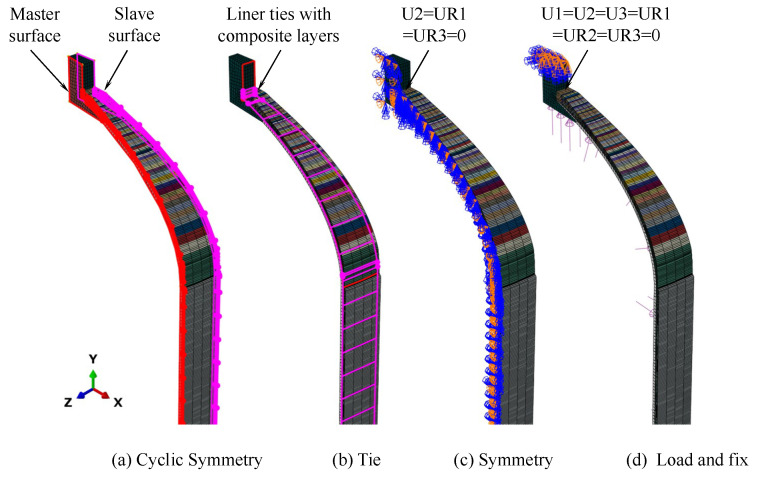
Loads and boundary conditions for composite shell.

To simulate the loading condition more realistically, the following loads and boundary conditions were applied in the finite element analysis, with reference to the shell arrangement during hydrotesting, as shown in Figure 3: periodic boundary conditions were applied to the cross-section of the 1/36 model and symmetrical constraints were applied to the main surfaces; the metal joints, rubber lining, and composite layer were connected using binding constraints; fixed constraints were applied to the end faces of the metal joints; and uniformly distributed loads were applied to the inner surfaces of the rubber lining and metal joints.

### 3.2. Shell Damage

**Figure 4:**
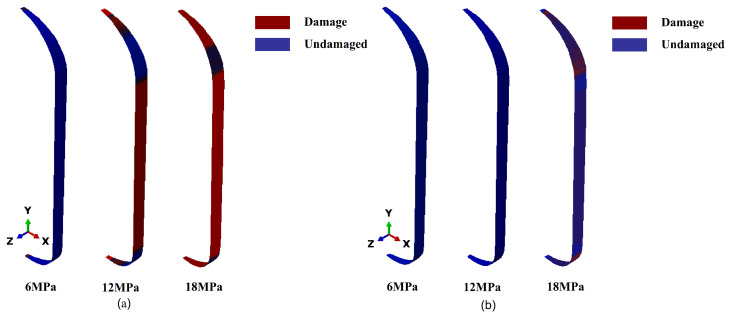
Damage results of unreinforced shell: (**a**) matrix failure; (**b**) fiber failure.

The hashin failure criterion was adopted. A fortran subroutine was written to determine failure, and the customized output of the ABAQUS results was implemented via the UVARM interface, using ABAQUS version 2017 (Dassault Systèmes, Vélizy-Villacoublay, France). The damage analysis results for the unreinforced shell under typical working conditions are shown in Figure 4.

As the internal pressure increased, matrix damage gradually propagated from localized regions to the entire structure. When the internal pressure reached 18 MPa, fiber damage was primarily concentrated in the dome’s equatorial region and the contact area with the metal boss. These regions were prone to localized stress concentrations due to geometric discontinuities and abrupt changes in stiffness, making them the main contributors to early structural failure.

Additionally, due to the absence of reinforcement layers, the hoop layers in the cylindrical section failed to bear the load effectively, and early damage in the dome region hindered effective axial load transfer, further reducing the overall pressure-bearing capacity of the structure.

**Figure 5:**
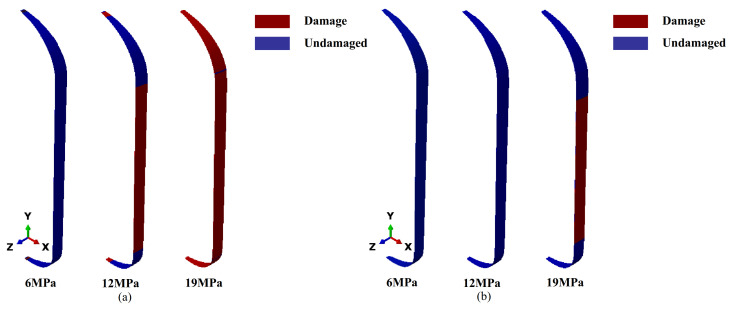
Damage results of five-layer reinforced shell: (**a**) matrix failure; (**b**) fiber failure.

For the five-layer reinforced shell, the damage response is shown in Figure 5. Under an internal pressure of 12 MPa, the matrix in the dome region remained largely intact, indicating that the reinforcement layer effectively delayed the initiation and propagation of early matrix cracks. As the internal pressure increased to 19 MPa, extensive matrix failure occurred in both the barrel and dome sections, while fiber failure was primarily concentrated in the barrel section.

The reinforcement layers not only improved local structural integrity but also modified the internal stress field by reducing peak stresses at the head–cylinder junction and more evenly redistributing hoop stresses across the shell. This delay in head-region failure allowed the cylindrical section to participate more fully in load-bearing, thereby shifting the failure mode closer to the designed burst location.

Comparative analysis of the two shells showed that the unreinforced shell failed before reaching the design pressure, with failure primarily attributed to early localized damage in structurally sensitive regions of the dome. This early failure deviated from the design goal of a cylinder-dominated burst. The complex curvature and interface geometry of the dome resulted in uneven stress transfer paths, making localized dome reinforcement essential. Although the five-layer reinforcement scheme effectively increased the load-bearing capacity and shifted the failure mode toward the cylindrical section, it introduced the issue of excess structural mass. Future designs should optimize the reinforcement layout by accurately identifying high-stress regions to achieve an optimal balance between structural strength and weight efficiency.

### 3.3. Experiment

**Figure 6:**
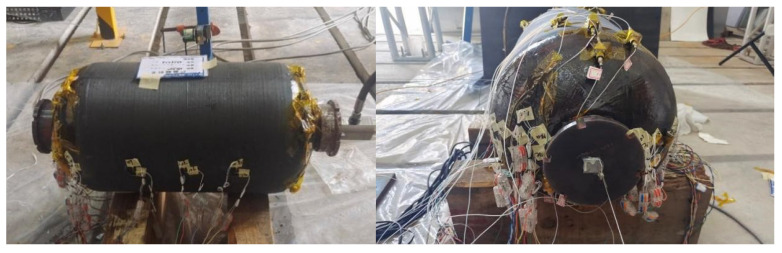
Water pressure test of composite shell.

The composite shell head region was fully reinforced with five layers of woven fabric, followed by hydrostatic testing [22,23,24]. The specimen was fabricated based on the designed lay-up schedule, and the test setup is shown in Figure 6. The test was conducted at room temperature (23 ± 1 °C) under quasi-static loading conditions, with pressure applied in steps of approximately 2 MPa every 30–60 s, reaching a maximum pressure of 16.7 MPa, held for 60 s before unloading.

**Figure 7:**
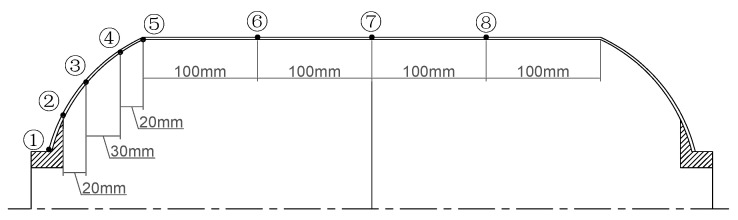
Distribution of strain gauge measurement points in the hydrostatic test.

In order to monitor strain responses in both the fiber-aligned and transverse directions during loading, strain gauges were installed on the surface of the composite shell. The measurement points were distributed across both the head and barrel sections and were arranged symmetrically along the axis of symmetry to ensure representative data collection. The specific locations of the strain gauges, numbered 1 through 8, are illustrated in Figure 7. Before testing, all strain gauges were calibrated using a standard preload and zero-adjustment process. Each test was conducted three times under identical conditions to ensure consistency. The maximum measurement uncertainty was estimated at ±2%, considering sensor accuracy and test consistency.

## 4. Results and Discussion

### 4.1. Comparison of Simulation and Experiment

**Figure 8:**
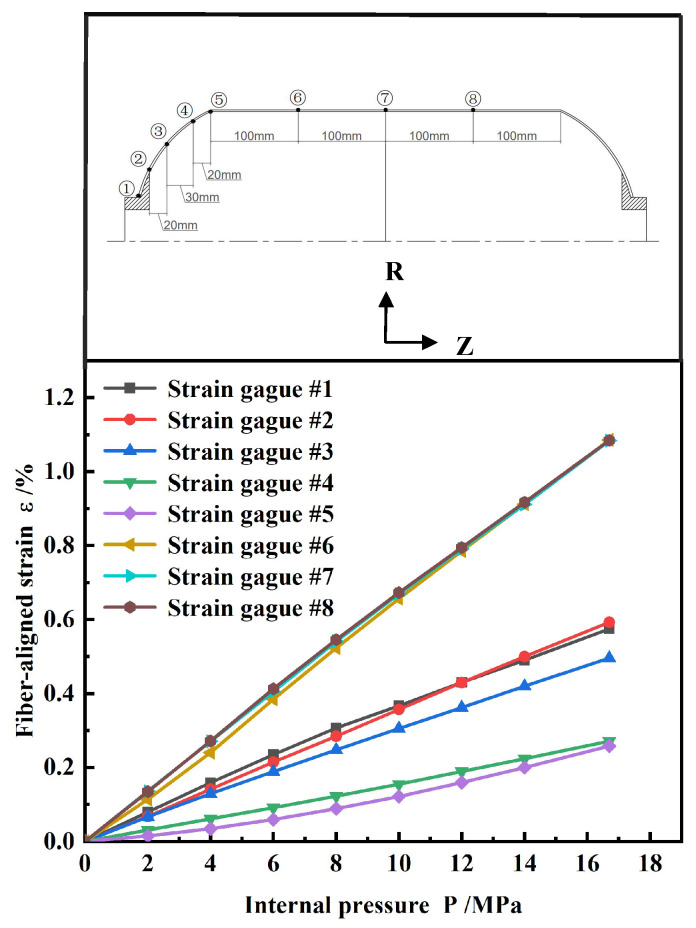
Load–strain curve.

Figure 8 shows the relationship curve of fiber-aligned strain with internal pressure at 16.7 MPa. The strain increase was linear, with no obvious nonlinear characteristics observed. This indicated that the shell maintained good structural integrity under high-pressure conditions. Moreover, strain in the head section was significantly lower than in the barrel section, verifying the effectiveness of the reinforcement structure in reducing local strain.

Previous tests have shown that damage to unreinforced structures mainly occurs at the interface between the metal head joint and the composite layer. Common failure modes include fiber breakage due to stress concentration at the interface and detachment of the metal joint from the composite layer under shear stress. This type of failure weakens the local load transfer capability and exacerbates overall structural degradation. This test further verified the effectiveness of the head reinforcement structure in enhancing the load-carrying capacity of the metal joint shoulder. Under low-pressure blasting conditions, the reinforcement structure effectively inhibited interface damage in the head section and prevented the metal joint from detaching or being ejected, improving the safety and integrity of the structure.

**Figure 9:**
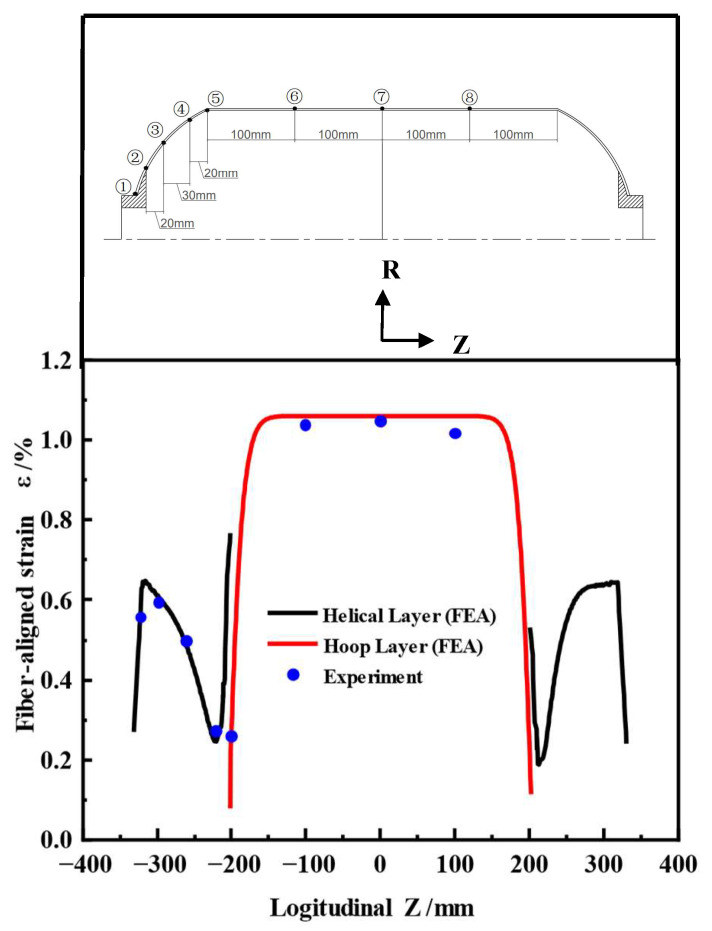
Comparison of fiber directional strain between experiment and analysis (16.7 MPa).

Figure 9 compares the fiber-aligned strains measured from the tests at 16.7 MPa water pressure with the finite element simulation results. Since the strain gauges were attached to the shell surface, the measured values correspond to the strain distribution in the outermost fiber layer. The black curve indicates the strain of the helical winding layer in the head section, while the red curve indicates the strain of the hoop winding layer in the barrel section.

Overall, the test values were in good agreement with the simulation results. The point with the largest deviation in the head section was point 4, showing an error of 5.9%. This point was located near the equator, which was the geometrical transition region from the cylinder section to the head section. The sudden change in local structure led to stress redistribution. The lack of hoop winding layer support in this region, along with the abrupt local stiffness change, limited the deformation, leading to a small strain value at this point. The maximum error in the barrel section occurred at point 8, with a 4% error, but the error at its symmetric position, point 6, was only 2%. Although the structure was symmetrical, the error still differed, which may have been related to measurement errors (such as strain gauge placement, contact state, or environmental effects). Manufacturing deviations, such as local layup deviation or resin enrichment, could lead to stiffness inconsistencies and cause deviations. Despite the local errors, the overall errors were within the engineering allowable range, confirming the accuracy and reliability of the simulation results.

### 4.2. Optimized Design of Finite Element Modeling

**Figure 10:**
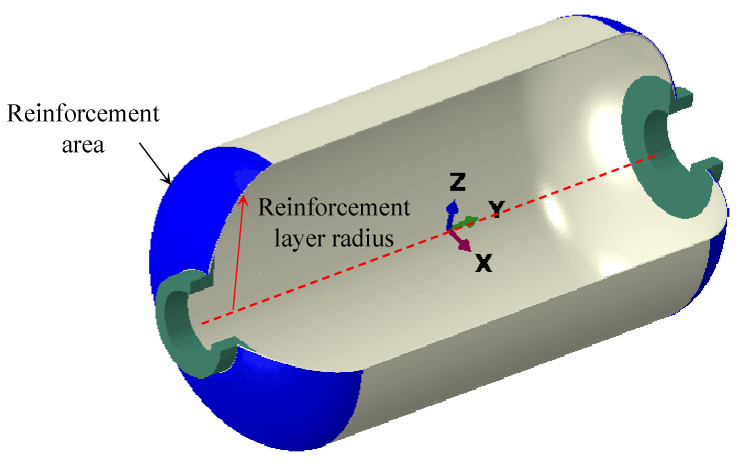
Schematic diagram of the reinforcement of the shell.

Traditional finite element simulation faces issues such as long computational cycles and high costs in structural optimization design. To address these problems, this paper introduces the surrogate modeling method. The agent model can predict the original model’s response with low computational resources, enabling the quick evaluation of different design parameter combinations and improving optimization efficiency. First, an experimental design was carried out before optimization. Then, the neural network agent model was constructed by combining approximate model optimization theory [25,26,27]. Based on engineering practice in fiber wound shells, the starting radius, number of reinforcement layers, and fiber bandwidth were selected as design variables. T700 carbon fiber braided fabric was selected as the reinforcement material, with a single layer thickness of 0.2 mm. The schematic diagram of shell reinforcement is shown in Figure 10.

Table 5 lists the range of values for the design variables. These parameters significantly affected structural performance. The starting radius of reinforcement affected both stress distribution and material usage in the head region; the number of reinforcement layers impacted the balance between the strength and weight of the shell; and the bandwidth determined winding efficiency and the uniformity of stress distribution. Selecting the optimal combination of parameters was crucial for structural performance optimization.

To construct the surrogate model, 30 design points were generated using Latin hypercube sampling [28,29] (LHS) within the defined design space. These were used to compute the maximum fiber-aligned stress in the dome region via finite element simulation, and the resulting dataset was used to train a neural network model.

The constructed neural network adopted a 3-5-1 architecture: three input neurons (corresponding to the three design variables), five neurons in a single hidden layer, and one output neuron that predicted the system response. To rigorously evaluate model performance, the dataset was randomly split into training, validation, and testing subsets at a 70%:15%:15% ratio, ensuring stratified coverage across all parameter ranges.

**Figure 11:**
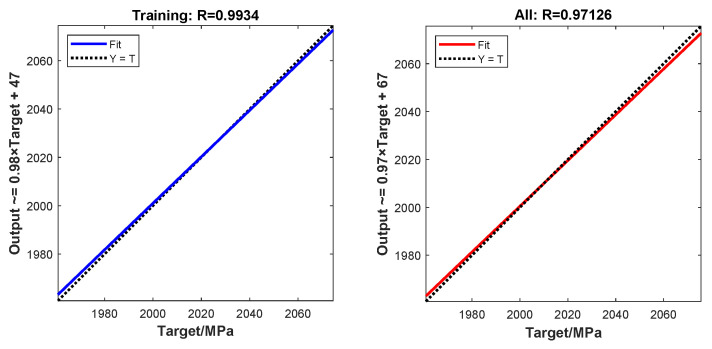
Neural network training results.

The left panel of Figure 11 shows the neural network’s fit to the training set, with a correlation coefficient of 0.99, indicating that the model accurately captured the underlying patterns in the training data. The correlation analysis of the overall dataset is shown in the right panel of Figure 11, with a correlation coefficient of 0.97, further validating the model’s strong fit and generalization capability.

To further evaluate the generalization capability of the surrogate model, we employed additional winding configurations that were not included in the original dataset. These configurations served as unseen FEA simulation cases to assess the predictive performance of the model. As shown in Table 6, the predicted fiber-aligned stresses were compared with the corresponding FEA results, and the relative errors were found to be 0.23%, 0.11%, and 0.19%, respectively. The results indicated that the surrogate model maintained a reasonable level of accuracy when applied to new input conditions.

Considering the complexity of the multi-objective optimization task, the NSGA-II algorithm [30,31] was adopted due to its proven capability to handle non-linear objectives and ensure a diverse and well-converged Pareto front. In this study, two competing objectives were considered: minimizing fiber-aligned stress and minimizing reinforcement volume in the dome region. The reinforcement starting radius R, number of reinforcement layers n, and fiber bandwidth w were selected as design variables, each constrained within feasible manufacturing ranges.

(6) $$\begin{array}{c} MinimizeF(x)=[FiberStress(R,n,w),Volum(R,n,w)]\\ 52\leq R\leq 69\\ 1\leq n\leq 5\\ 6\leq w\leq 12 \end{array}$$

**Figure 12:**
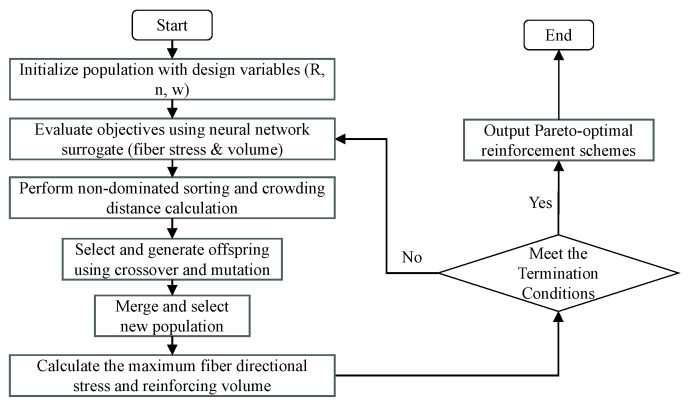
Genetic algorithm optimization process.

F(x) is the fitness function. The neural network surrogate model was used to evaluate objectives efficiently during the optimization. Figure 12 illustrates its optimization process.

**Figure 13:**
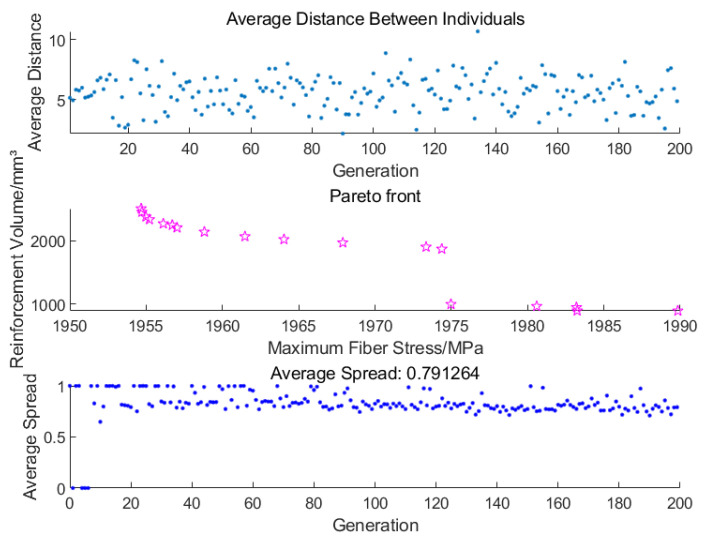
Optimization results for reinforcement.

Figure 13 illustrates the results of the head reinforcement optimization process. The average distance of the population individuals in each generation (blue dots) fluctuates between 5 and 10 without any significant decrease, indicating that the population diversity remains high, which helps to avoid premature convergence. The Pareto front for objective 1 (fiber stress) and objective 2 (reinforcement volume) is uniformly distributed with good coverage, where the pink stars represent the non-dominated solutions obtained during the optimization process. This demonstrates that the algorithm achieves a satisfactory trade-off between multiple objectives. The average spread in each generation (also shown with blue dots) stabilizes at 0.8, suggesting that the solution set exhibits both good coverage and diversity.

The objective of this study was to achieve reinforcement lightweighting while maintaining structural strength. The results show that when R = 67 mm, n = 1 layer, and w = 7 mm, the optimal solution met the strength requirement (stress < 2000 MPa) and effectively minimized the reinforcement volume. Table 7 lists the specific parameters of this design combination.

**Figure 14:**
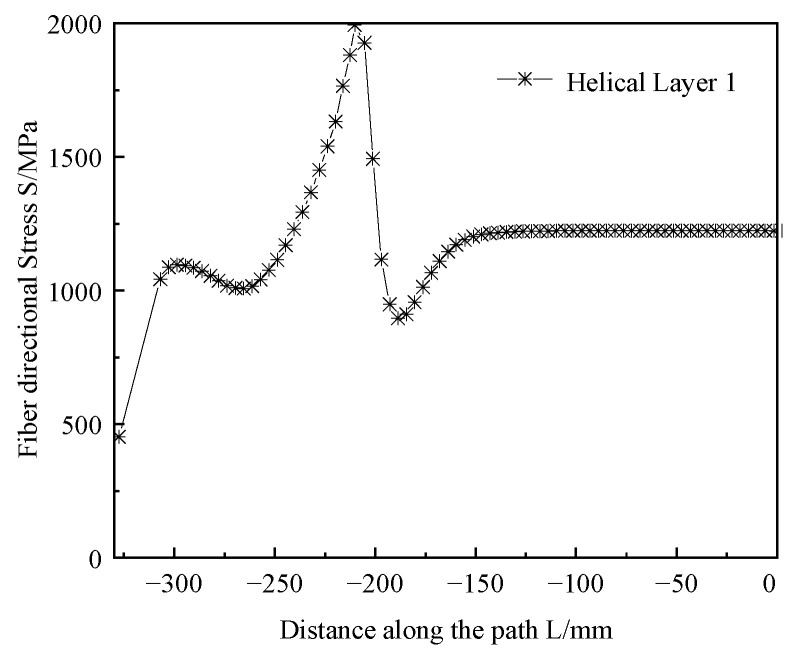
Optimized shell structure dome fiber direction stress.

**Figure 15:**
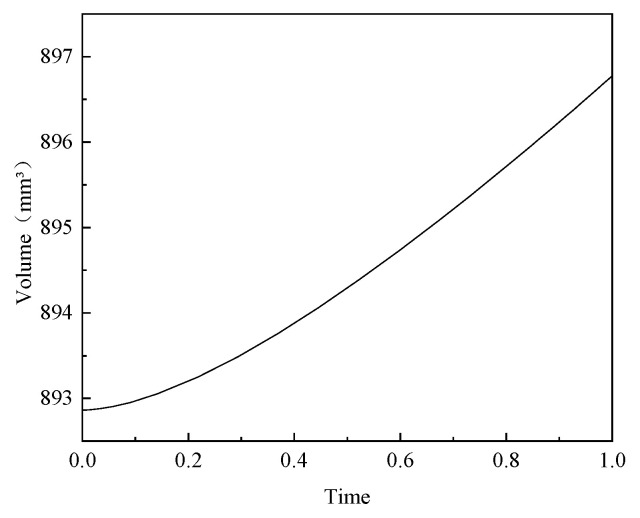
Optimized shell structure dome reinforcement volume.

Given that the results of the optimization algorithm depended on the predictive performance of the surrogate model, the accuracy of the resulting optimal solution required validation. We applied the optimized parameter combinations to the finite element model for solving to evaluate the results of the optimized design. The simulation results are shown in Figure 14, with the maximum fiber-aligned stress of the head being 1993.15 MPa. Figure 15 shows a head reinforcement volume of 892.86 mm^3^ (1/36 of the overall model). Compared with the predicted values of the surrogate model, the maximum stress error was 0.17% and the reinforcement volume error was 0.29%. This result indicated that the constructed surrogate model had high prediction accuracy and reliability. After optimization of the shell structure, while ensuring that the maximum fiber-aligned stress in the head was less than 2000 MPa, the overall mass was calculated to be reduced from 2.641 kg to 2.396 kg, a reduction of 9.3%. This result proves the effectiveness of multi-objective optimization in optimizing the carbon fiber composite shell structure.

**Figure 16:**
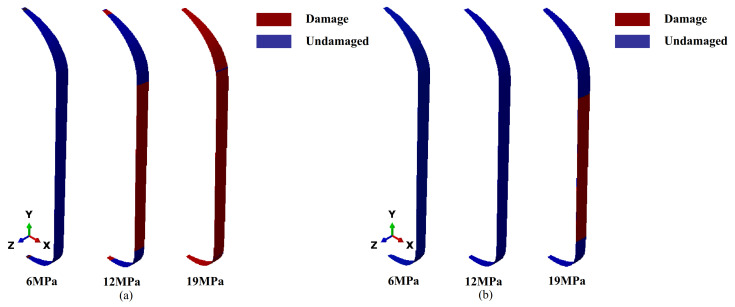
Damage results of optimized reinforced shellÑ (**a**) matrix failure; (**b**) fiber failure.

Finally, the optimal parameters were applied to structural damage analysis. The simulation analysis is shown in Figure 16. Figure 16a shows that the matrix damage first occurred in the cylinder body section at 12 MPa. With further loading, damage developed in the head region. This phenomenon was primarily due to the higher hoop stresses applied to the cylinder body, causing the matrix material to exhibit tensile damage first. As the load continued to increase, the stress in the head region rose, eventually causing failure of the matrix material there as well. Figure 16b presents the simulation results of fiber failure, indicating that the fibers in the cylinder body section first failed over a large area, reflecting the critical state of structural damage under a 19 MPa internal pressure. This result strongly aligns with the phenomenon observed in the actual hydraulic bursting test, demonstrating the accuracy and reliability of the optimized solution.

## 5. Conclusions

This study focused on the structural reinforcement and optimization of composite shells subjected to low-pressure explosive loads. To address the issue of premature failure at the dome section due to stress concentration, a localized reinforcement strategy was proposed and validated. Through numerical simulations, experimental verification, and multi-objective optimization, the following key conclusions were drawn:

(1) The reinforcement structure effectively shifted the failure location to the cylindrical section, enhancing the load-bearing capacity of the fibers. It also significantly alleviated stress concentration at the joint shoulder, thus preventing premature failure under low-pressure conditions.
(2) A refined finite element model of the head was developed, incorporating variable angles and thicknesses. Comparison with hydrostatic test results showed deviations of 4% at the center of the cylindrical section and 5.9% at the joint shoulder, both within acceptable engineering limits, confirming the model’s precision and reliability.
(3) A high-precision surrogate model was developed using Latin hypercube sampling and neural networks, achieving a correlation coefficient of 0.99 and a prediction error below 0.29%. The model significantly reduced computational costs and enhanced the efficiency and stability of the multi-objective optimization process.
(4) An optimization scheme was proposed based on the NSGA-II algorithm, aiming to minimize both the maximum stress on the head and the volume of the reinforcement. This approach enabled a lightweight design while ensuring the required mechanical performance.

## Figures and Tables

**Table 1:** Ply schemes to design burst pressure of composite shells.

Design Burst Pressure	Hoop Layers	Helical Layers	Ply Schemes
19 MPa	6	6	[±19.5/90/±19.5/90_4_]

**Table 2:** Composite shell winding process.

Layer Number	Wind Angle/°	Layer Type	Band Width/mm
Dome reinforcement 2 times			
1	+19.5°	Helical	8.8
2	−19.5°	Helical	8.8
Dome reinforcement 2 times			
3	90°	Hoop	8.1
4	+19.5°	Helical	8.8
5	−19.5°	Helical	8.8
6	90°	Hoop	8.1
Dome reinforcement 2 times			
7	+19.5°	Helical	8.8
8	−19.5°	Helical	8.8
9	9°	Hoop	8.1
10	90°	Hoop	8.1
11	90°	Hoop	8.1
12	90°	Hoop	8.1

**Table 3:** Mechanical properties of liner and metal boss.

Parameter	Liner	Metal Boss
Elasticity modulus *E*/GPa	0.0078	200
Poisson’s ratio/μ	0.47	0.3

**Table 4:** Mechanical properties of T800 carbon fiber composite.

Parameter	Value
Tensile modulus $E_{11}$/GPa	161
Tensile modulus $E_{22}$/GPa	8.82
Tensile modulus $E_{33}$/GPa	8.82
Shear modulus $G_{12}$/GPa	5.33
Shear modulus $G_{23}$/GPa	2.74
Shear modulus $G_{13}$/GPa	5.33
Poisson’s ratio $\mu_{12}$	0.33
Poisson’s ratio $\mu_{23}$	0.45
Poisson’s ratio $\mu_{13}$	0.33
Longitudinal tensile strength $X_{t}$/MPa	2100
Longitudinal compressive strength $X_{c}$/MPa	1400
Transverse tensile strength $Y_{t}$/MPa	60
Transverse compressive $Y_{c}$/MPa	150
Shear strength *S*/MPa	70

**Table 5:** Optimization variable parameter value range.

Parameter	Range
Reinforcement start radius	52–69
Reinforcement layers	1–5
Bandwidth	6–12

**Table 6:** Comparison of predicted and FEA values for additional winding methods.

Winding Method	Predicted Value/MPa	Finite Element Value/MPa	Error/%
[55 4 9]	2060.32	2065.06	0.23
[60 1 7]	1993.16	1995.35	0.11
[65 3 6]	2048.07	2051.96	0.19

**Table 7:** Optimal adaptation parameters.

Parameter	Value
Reinforcement start radius	67
Reinforcement layers	1
Bandwidth	7
Maximum fiber-aligned stress/MPa	1989.85
Reinforcement volume/mm^3^	890.31

## Data Availability

The raw/processed data required to reproduce these findings cannot be shared at this time as the data also form part of an ongoing study.
